# Designing healthier neighbourhoods: a systematic review of the impact of the neighbourhood design on health and wellbeing

**DOI:** 10.1080/23748834.2020.1799173

**Published:** 2020-09-01

**Authors:** Janet Ige-Elegbede, Paul Pilkington, Judy Orme, Ben Williams, Emily Prestwood, Daniel Black, Laurence Carmichael

**Affiliations:** a Centre for Public Health and Wellbeing, University of the West of England, Bristol, UK; b Air Quality Management Resource Centre, University of the West of England, Bristol, UK; c Daniel Black + Associates | db+a, Bristol, UK; d WHO Collaborating Centre for Healthy Urban Environments, University of the West of England, Bristol UK

**Keywords:** Neighbourhood design, health and wellbeing

## Abstract

Several studies have investigated the impact of neighbourhood design on health and wellbeing, yet there are limited reviews investigating the quality of the evidence and the most effective interventions at a population level. This systematic review aims to clarify the impact of the neighbourhood design on health and wellbeing and evaluate the quality of the evidence underpinning such associations. Eight electronic databases were searched for studies conducted between 2000 and 2016. Additional searches were conducted on Google to identify potentially eligible grey literature. A total of 7694 studies were returned from the literature search, and a final selection of 39 studies were deemed eligible for inclusion. Quality appraisal was conducted using the Quality Assessment Tool for Quantitative Studies. Findings from the studies showed important associations between neighbourhood design principles such as walkability, access to green space and amenities on health and wellbeing. Findings from this review also highlight areas with inconsistent findings and gaps in the evidence for future research.

## Introduction

As research into the impact of neighbourhood environment on health advances (Renalds *et al*. 2010, Barton *et al*. 2015, Public Health England 2017, Smith *et al*. 2017), it is essential to evaluate the strength and quality of the evidence to identify the most effective interventions and understand the mechanism underpinning such interventions. Such mechanisms are likely to differ depending on the characteristics of a population. This review aims to fill this gap by providing a thorough assessment of the strength and quality of the evidence. Findings from this review can provide local policymakers with a range of evidence-based interventions about aspects of the neighbourhood environment that will have the greatest impact on health and wellbeing of specific population groups. The study also provides the basis for an economic evaluation of the impact of neighbourhood design on health and wellbeing. This research is part of a larger UPSTREAM project that aims to investigate the barriers and opportunities for integrating health and wellbeing into upstream urban development decision-making (Black *et al*. 2018)

Neighbourhoods are places people dwell, work and have a sense of belonging (Bird *et al*. 2017). The environments and neighbourhood people live in can have a profound impact on their health and wellbeing (Dannenberg *et al*. 2011, Bird *et al*. 2018). Neighbourhood design that promotes a healthy lifestyle can improve the health and wellbeing of residents (Lees *et al*. 2014). Street connectivity, land use mix and access to amenities and services are essential features of good neighbourhood design. A poorly designed neighbourhood adversely affects the health and wellbeing of everyone living in it (Public Health England 2017).

Besides, three important features of neighbourhood design: completeness, compactness, and connectivity are essential for promoting healthy behaviours (Blackson 2012). A complete neighbourhood is one that maximises land use to cater for a range of activities (including business, social, and religious activities) to meet the requirements of people living in the area (The Young Foundation 2010, Barton *et al*. 2015). The compactness of a neighbourhood refers to the situation of places within walking distances to amenities and facilities, while connectivity not only deals with public transport options that connect neighbourhoods but also encompasses opportunities for social connectedness within neighbourhoods. Higher-density development in which a variety of land uses are located such that residents and workers are within walking distance of many destinations are likely to promote social interaction (Lees *et al*. 2014, Bird *et al*. 2017).

The impact of the neighbourhood environment on health can be felt across the life course (Villanueva *et al*. 2013, Gustafsson *et al*. 2013, 2014). Evidence from longitudinal studies suggests that living in poorly designed neighbourhoods with high level of neighbourhood deprivation, neighbourhood crime, and poor housing condition can significantly increase the risk of low birth weight (O’campo *et al*. 1997, Schempf *et al*. 2009) and can affect health and wellbeing of adolescents (Boardman and Saint Onge 2005, Villanueva *et al*. 2013). Children are highly influenced by their neighbourhood environment. Barriers to physical activity at the neighbourhood level can influence a child’s long-term behavioural pattern (Fiechtner *et al*. 2015). Several aspects of neighbourhood design including the presence of public open space and neighbourhood connectivity can optimise opportunities for social interactions (Beard and Petitot 2010) and address social issues such as loneliness among older adults (Ige *et al*. 2019).

Evidence from several systematic reviews investigating aspects of the built environment that impact on health and wellbeing reiterate the importance of neighbourhood walkability (Renalds *et al*. 2010) and infrastructural improvements including access to open space (Smith *et al*. 2017) on inequalities, behavioural and health outcomes. These reviews and indeed other existing reviews (Van Cauwenberg *et al*. 2011, Twohig-Bennett and Jones 2018) provide useful evidence; however, the findings are limited to specific health outcomes arising from selected aspects of neighbourhood design. There is a dearth of systematic review that examines all possible health outcomes arising from the design of the neighbourhood. Such evidence is needed to provide a holistic overview of the range of health outcomes associated with neighbourhood design across the life course. This study aims to systematically review the impact of neighbourhood design on health and wellbeing. In addition to the aforementioned aim, this study also provides the basis for subsequent economic evaluation of the impact of neighbourhood design on health and wellbeing.

## Methods

### Search strategy

The decision to focus on the neighbourhood design stems from a broader mapping exercise of the key features of the built environment that impacts health and wellbeing. This mapping exercise was conducted using the Barton and Grant (2006) health map and the Public Health England Spatial Planning for Health Tool (Public Health England 2017). An initial scoping exercise was performed on Google scholar to compile a list of databases from previous reviews across similar areas (Durand *et al*. 2011, Mackenbach *et al*. 2014). The scoping exercise enabled the identification of search terms. The search terms were categorised into three-word groups relating to the characteristic neighbourhood design, health outcomes and study type. Following an initial draft of search terms, subject area experts were contacted to verify and refine the terms. A pilot search was performed by the project researcher in one database (MEDLINE) to test the search strategy and refine the search terms before the full search was undertaken. A structured search for published literature was conducted by the project researcher across eight electronic databases (MEDLINE, PsychINFO, Cumulative Index to Nursing & Allied Health Literature, Applied Social Sciences Index and Abstracts, Cochrane Database of Systematic Reviews, SocINDEX, Econlit, Allied and Complementary Medicine) to identify relevant publications from January 2000 to November 2016. Additional searches were conducted on google and google scholar to locate potentially eligible studies and grey literature. This was combined with hand-searching of reference lists. All authors were involved in identifying relevant literature. This study was conducted in accordance with the Preferred Reporting Items for Systematic Reviews and Meta-Analyses (PRISMA) checklist (Liberati *et al*. 2009, Swartz 2011).

### Eligibility

Studies were eligible for inclusion in the review if they met the following criteria: (1) report on measurable associations between health outcomes (primary or secondary) and any characteristics of neighbourhood design. (2) are published in English language between January 2000 to November 2016 with full text in a peer-reviewed journal or nationally recognised stakeholder website. (3) are conducted in a high-income country according to the World Bank categorisation (World Bank 2017).

Qualitative studies were excluded from this review as the focus on identifying any measurable impact on health outcomes of the neighbourhood environment on health precludes the inclusion of qualitative variables. Also, the quantitative results from this study formed the basis for the development of an economic modelling exercise reported elsewhere (UPSTREAM 2018).

All studies retrieved from the search database were exported to RefWorks for duplicate removals. Studies were screened by titles, abstract and full text against the inclusion and exclusion criteria. Two reviewers (J.I. and P.P.) independently assessed the quality of selected studies and extracted relevant data.

### Data extraction

A standardised data extraction tool was created on Microsoft Word to report key characteristics and findings from eligible studies. Information about the author(s), year of publication, location of study, variable of interest relating to neighbourhood design, characteristics of the study population, key findings, and quality rating were all extracted unto the data extraction sheet.

### Quality appraisal

Quality appraisal was performed using the Quality Assessment Tool for Quantitative Studies by the Effective Public Health Practice Project (EPHPP). This tool has received good recommendations based on construct validity and acceptable content (Jadad *et al*. 1996, Mulrow *et al*. 1997) and has been used for similar reviews (Chillón *et al*. 2011, Fitzpatrick-Lewis *et al*. 2011, Ige *et al*. 2018). The tool consists of six quality assessment domains: The probability that the study participants are representative of the target group (selection bias); the design of the study; the control of confounding factors; the concealment of participants and researchers (blinding); the reliability and validity of data collection methods; reporting of withdrawals and dropout rate. (Mulrow *et al*. 1997, Thomas *et al*. 2004, Jackson and Waters 2005). Each component includes a standardised set of questions and answers to determine the component quality rating as strong, moderate or weak. The overall quality rating for each study was determined as strong, moderate or weak based on the rating of the six components. Studies with no weak rating for any of the six components were rated strong, studies with only one weak rating for any of the six components were rated moderate while studies with more than one weak rating for any of the six components were rated weak.

## Results

**Figure 1.**
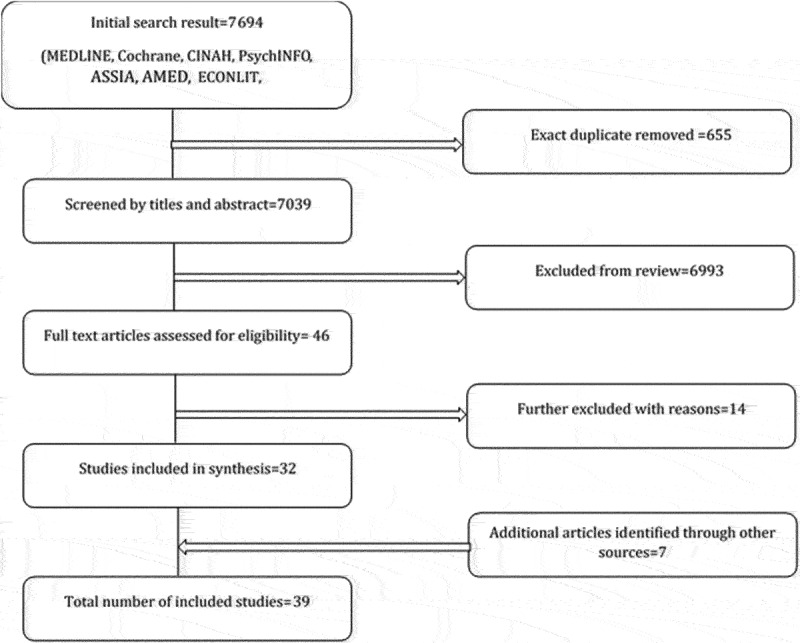
Study selection process for neighbourhood design.

Our search database returned a total of 7694 studies. Duplicates were removed, leaving a total of 7039 studies. These studies were screened for eligibility by titles and abstracts, followed by full-text screening. A final selection of 39 studies was included in the review. Over a quarter (n = 11) of included studies were cross-sectional studies with limited sample size. Eight of the included studies were cohort studies, two were longitudinal studies, seven were quasi-experimental studies and the rest included other study designs. Over 40% of the included studies (n = 17) were rated as weak quality based on study design and methodological rigour. These studies were excluded from the final analysis. The final analysis reported in this review comprises of 22 studies deemed to be of moderate (n = 13) and strong (n = 9) quality. Seven of these were conducted in the United States; two were conducted in Canada, and nine studies were conducted in other parts of Europe including the UK. Two studies were conducted in Australia, and one study was conducted in New Zealand. Figure 1 shows a detailed breakdown of the search strategy

## Findings of the review

**Figure 2.**
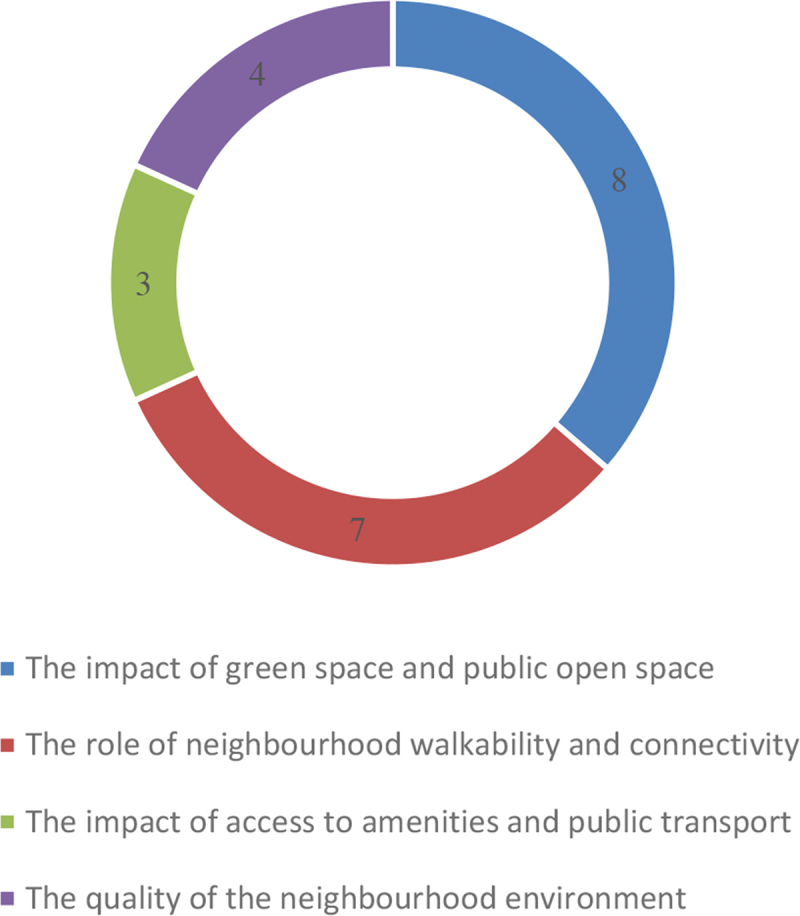
Breakdown of studies included in the review.

Eight of the 22 studies analysed in this review discussed the impact of green space and public open space on health and wellbeing while seven studies examined the role of neighbourhood walkability and connectivity on health and wellbeing. Access to amenities and transport facilities was discussed in three studies, while four studies investigated the impact of neighbourhood quality on health and well-being. (Figure 2).

### The impact of green space and public open space

The findings from the studies listed under this category showed potential benefits of green space on behavioural outcomes such as increased physical activity (Picavet *et al*. 2016; Sugiyama *et al*. 2010) and on reduction of mortality (Villeneuve *et al*. 2012, Mueller *et al*. 2016) and risk factors for cardiovascular diseases (Paquet *et al*. 2014, Tamosiunas *et al*. 2014). However, negative associations were reported between green space and risk of asthma (Andrusaityte *et al*. 2016) and findings were inconclusive for mental health (Annerstedt *et al*. 2012) (Table 1).

**Table 1.** Main findings from studies on the impact of green space and public open space.

First author, year	Study design^a^	Population	N/hood design Indicator	Health outcome(s)	Location(s)	Main findings	Quality of study
Andrusaityte *et al*. 2016	Case-C	Children 4–6 years N = 1489	Neighbourhood greenness measured objectively using a standardised protocol	Asthma measured by response of parents to standardised asthma questionnaire	Lithuania	Surrounding greenness was measured using an average of satellite-based normalised Difference Vegetation Index (NDVI) within buffers of 100 m, 300 m and 500 m from home address of participants. Increase in the NDVI in buffers of 100, 300 and 500 m was associated with higher risk of asthma. An IQR increase in NDVI-100 m significantly increased risk of asthma (OR 1.43, 95% CI 1.10 to 1.85). 100≤ median and the distance to a city park >1000 m (OR 1.47, 95% CI 0.56 to 3.87). The pattern of associations was similar but not statistically significant in the adjusted models. Distance to parks was also not significantly associated with increased risk of asthma in both adjusted and unadjusted models	M
Villeneuve *et al*. 2012	Cohort	Adults ≥35 years N = 575,000	Distance to urban green space defined using the Landsat satellite and NVDI	Mortality	Canada	The authors found that an increase in the interquartile range (IQR)of green space, using a 500 m buffer, was associated with a decline in rate of non-accidental mortality (RR = 0.95, 95%CI = 0.94–0.96). Reductions in mortality with increased residential green space was observed for each underlying cause of death with the strongest association in the order; respiratory disease (RR = 0.91, 95%CI = 0.89–0.93), stroke (RR = 0.95, 95%CI = 0.92–0.97) and Ischemic heart disease (RR = 0.95, 95%CI = 0.94–0.97). Estimates were unchanged after adjusting for ambient air pollution	S
Tamosiunas *et al*. 2014	Cohort	Adults 45–72 years N = 5112	Accessibility of urban green space measured using geo-coding software. Green space was defined as city parks larger than one hectare	Cardiovascular diseases measured objectively using laboratory analysis of cholesterol, blood. Anthropometric measurement was also assessed alongside self-reported data obtained via questionnaire	Lithuania	Result showed that the prevalence of cardiovascular risk factors and prevalence of diabetes was significantly lower among park users than non-park users. However, the increased risk of CVD in relation to the accessibility of green spaces was only statistically significant among men and not women. Residential distance to green spaces was not associated with prevalence of any health-related variable of interest. Hazard ratio1.36 95%CI = 1.03–1.80 Compared to non-users, park users were less likely to smoke regularly(OR = 0.82 95%CI = 0.69–0.97, P = 0.001, X^2^ = 14.6), be obese(OR = 0.75, 95%CI = 0.64–0.84, P = 0.001) and physically inactive (OR = 0.74, 95%CI = 0.64–0.84, P < 0.001 X^2^ = 21.1), to have high levels of fasting glucose (≥ 7.0 mmol/L) OR = 0.67, 95%CI = 0.55–0.83, P = 0.004 X^2^ = 10.9), have poor self-rated health (OR = 0.69 95%CI = 0.56–0.83, P = 0.036 X^2^ = 6.63) and quality of life (OR = 0.63, 95%CI = 0.46–0.85, P = 0.012, X^2^ = 8.82) and had a lower prevalence of diabetes mellitus (OR = 0.72, 95%CI = 0.58–0.90, P = 0.031, X^2^ = 4.65).	S
Mueller *et al*. 2016	HIA	Barcelona residents ≥20 years Estimates were based on data from 1,357,361 residents	Access to green space, physical activity, air pollution, noise and heat. These variables were measured using existing WHO and European Commission guidelines	Premature deaths, life expectancy and economic impacts	Spain	. Findings showed that 20% of annual mortality can be prevented by complying with international recommendations for performance of physical activity, exposure to air pollution, noise health and access to green space. An increase in physical activity was associated with highest decline in preventable deaths, followed by reduced exposure to air pollution and traffic noise. Compliance with international recommendations increased average life expectancy by 360 days (95%CI = 219–493) and resulted in economic savings of 9.3 (95%CI = 4.9–13.2) billion € per year.	M
Picavet *et al*. 2016	C-C	Adults (20–59 years) N = 4796	Green-space measured using satellite data	Physical activity and general health measured using questionnaire data, and examination by trained personnel	Netherlands	. Findings suggest that being active was not associated with the overall percentage of green but urban green pace was associated with more time spent cycling (β1 km = 0.17, 95%CI = 0.009–0.25) and sports (β1 km = 0.07, 95%CI = 0.02–0.13) and less time spent gardening (β1 km = −0.28, 95%CI = −0.36to −0.21) and doing odd jobs (β1 km = −0.20, 95%CI = −0.29 to −0.11). In contrast, more agricultural green was associated with less time spent cycling (β1 km = −0.15, 95%CI −0.13 to −0.04) and sports (β1 km = −0.04, 95%CI −0.07 to −0.01) and more time spent on gardening (β1 km = 0.16, 95%CI 0.12 to 0.19) and odd jobs (β1 km = 0.10, 95%CI 0.05 to 0.15). Green space within 1 km radius was associated with fewer depressive complaints (β = −0.27 95%ci = −0.42 to −0.11) and better physical functioning (0.29, 95%CI = 0.02 to 0.56).	S
Paquet *et al*. 2014	L	Adults≥18 years N = 3205	neighbourhood walkability, presence of Public Open Spaces (POS) assessed using GIS data	Incidence of pre-diabetes/diabetes, hypertension, dyslipidaemia and abdominal obesity	Australia	POS was categorised into size, type and greenness. The size of public open spaces was also associated with lower risk of developing prediabetes/diabetes (RR = 0.75 95%CI = 0.69–0.83 p < 0.0001). No significant associations were found for hypertension and dyslipidaemia	M
Sugiyama *et al.* 2010	Q	Adults 18 years or older intending to move to new home N = 1,366	Attractiveness, size and proximity too neighbourhood open spaces measured using GIS data	Recreational walking determined from self-reported data	Australia	Findings showed that shorter distance to attractive open spaces was associated with higher levels of recreational walking (OR = 1.38, 95%CI = 1.10–1.73, P < 0.01). However, adults with larger attractive open spaces within 1.6 km to their home reported higher odds of being able to walk 150 minutes or more in a week (OR = 1.39 95%CI = 1.08–1.79).	M
Annerstedt *et al*. 2012	Cohort	Adults 18–80 years (mean age = 50) N = 24,945	Green qualities in neighbourhood (serene, wild, lush etc.) assessed using GIS data	Mental health measured by responses to self-administered questionnaires	Sweden	The authors reported that mental health was not affected by access to green qualities investigated (Access to space OR (men) = 1.1, P = 1.74, OR (women) = 1.1, P = 0.54) (Access to serene OR (men) = 0.9, 95%CI = 0.5–1.6, P = 0.77, OR (women) = 0.8 95%CI = 0.5–1.2, P = 0.29). However, there was a significant interaction between physical activity and access to serene (OR = 0.2 95%CI = 0.06–0.9 P = 0.05)/access to space (OR = 0.3 95%CI = 0.1–0.9 P = 0.045) among women only.	S

Andrusaityte *et al*. (2016) found that proximity to green space measured using the Normalised Difference Vegetation Index (NDVI) was associated with asthma among children. The authors reported that the risk of asthma among 4-6-year-olds increased significantly by 43% with an interquartile increase in greenness within 100 m of residential address, while close residence to a city park was not statistically significantly associated with asthma risk. The cohort study by Villeneuve *et al*. (2012) reported that an increase in the interquartile range of green space was associated with a decrease in non-accidental mortality (RR = 0.95, 95%CI = 0.94–0.96). The association was strongest for reduced mortality from respiratory diseases (R = 0.91, 95%I = 0.89–0.93). Tamosiunas *et al*. (2014) conducted a cohort study to determine the associations between proximity to green space and the prevalence of cardiovascular diseases. The authors reported that residential distance to green space was not associated with any health-related variable of interest. However, the prevalence of cardiovascular risk factors and diabetes was found to be significantly lower among park users than non-park users. Also, compared to non-park users, park users were less likely to be obese (OR = 0.75, 95%CI = 0.64–0.84, P = 0.001) and had a lower prevalence of diabetes mellitus (OR = 0.72, 95%CI = 0.58–0.90, P = 0.031). A quasi-experimental study investigating the impact of features of green space on recreational walking found that proximity to attractive open space was associated with higher levels of recreational walking (OR = 1.38, 95%CI = 1.10–1.73, P < 0.01) (Sugiyama *et al*., 2010).

Mueller *et al*. (2016) in their health impact assessment to determine the number of preventable premature deaths associated with exposure to green space, reported that compliance with international exposure recommendations for access to green space, physical activity, air pollution noise and heat could prevent 20% of annual mortality. Findings from Picavet *et al*. (2016) showed that urban green space was associated with more time spent cycling and participating in sports and less time spent gardening and doing odd jobs. In contrast, agricultural green space was associated with less time spent cycling and participating in sports and more time spent gardening and doing odd jobs. A longitudinal study by Paquet *et al*. (2014) investigated the associations between the size of public open space and the incidence of prediabetes/diabetes, hypertension, dyslipidaemia, and abdominal obesity. The authors reported that one standard deviation increase in median NDVI was associated with a 25% lower risk of developing pre-diabetes/diabetes (RR = 0.75, 95%CI = 0.69–0.83, P < 0.0001). The mental health and behavioural impact of neighbourhood green qualities were investigated in the study by Annerstedt *et al*. (2012). There was no significant association between the neighbourhood green quality investigated and mental health. However, the authors reported a significant association between physical activity and access to serene neighbourhoods among women.

### The role of neighbourhood walkability and connectivity

Neighbourhood walkability was associated with a positive impact on mental health (Berke *et al*. 2007), reduced incidence of hypertension (Chiu *et al*. 2016), diabetes (Paquet *et al*. 2014), lower risk of disability (Freedman *et al*. 2008) and reduced air pollution (James *et al*. 2015). No significant association was reported on the impact of neighbourhood walkability on BMI (Sriram *et al*. 2016) while Mecredy *et al*. (2011) reported negative associations between walkability and physical activity. (Table 2).

**Table 2.** Main findings from studies on the role of neighbourhood walkability and connectivity.

First author, year	Study design^a^	Population	N/hood design Indicator	Health outcome(s)	Location(s)	Main findings	Quality of study
Berke *et al*. 2007	C-S	Older adults ≥65 years N = 740	Neighbourhood walkability measured by linking data from a previous longitudinal study with GIS data	Depression (measured by the Centre for Epidemiologic Studies Depression Scale)	USA	After adjusting for potential confounders, there was a significant association between neighbourhood walkability and depressive symptoms in men. Odds ratio for IQR of walkability score ranged from 0.31–0.33, P = 0.02. No significant association was observed for women in adjusted or non-adjusted models	M
Chiu *et al*. 2016	Cohort	Adults≥20 years N = 32,626	Neighbourhood walkability (defined as Walkscore)	Incidence of hypertension	Canada	There was a significantly lower risk of incident hypertension among people who moved from areas of low walkability-high walkability versus those who moved from an area of low walkability to another area of low walkability (hazard ratio = 0.46; 95% CI = 0.26–0.81 P < 0.01). The crude hypertension incident rates were 18.0 per 1000 person-years (95%CI = 11.6–24.8) among participants who moved from areas of low- low walkability and 8.6 per 1000 person-years (95%CI = 5.3–12.7) among those who moved from areas of low walkability-high walkability (P < 0.001). The authors reported no significant differences in the hazard of annual health examination between the two groups	S
James *et al*. 2015	C-S	Women 30–55 years N = 62,588	Neighbourhood walkability measured using census and GIS data	Air pollution (PM 2.5) measured using GIS-based spatiotemporal	USA	After adjusting for potential confounders, the highest tertile of walkability index 1.58 (95%CI1.54, 1.62), intersection count 1.20(95%CI1.16, 1.24), business count 1.31(95%CI 1.27, 1.35), and population density 1.84 (95%CI1.80, 1.88) was associated higher level of PM_2.5_ µg/m^3^ compared to the lowest tertile.	M
Sriram *et al*. 2016	C-S	Older women (63–99 years) N = 6,164	Neighbourhood walkability, measured as Walk Score	Adiposity and BMI	USA	After adjusting for potential confounders, there was no association between higher walk score and BMI or overall obesity. However, respondents living in highly walkable areas (i.e. with higher walk score) had significantly lower odds (OR = 0.72 95%CI = 0.53–0.99) of abdominal obesity (waist circumference >88 cm) when compared to counterparts in less walkable areas (OR = 1.04 95%CI = 0.91–1.18).	M
Mecredy *et al*. 2011	C-S	Students in grade 6–10 from 180 Canadian schools N = 8,535	Street connectivity measured using survey and GIS data	Self-reported physical activity levels	Canada	Street connectivity was measured using data from geographical information system. Physical activity outside the school was measured by self-reported questionnaire. The authors reported that compared to children living in the highest street connectivity quartile, those in the second (RR = 1.22, 95%CI = 1.10–1.35), third (RR = 1.25, 95%CI = 1.13–1.37) and fourth (RR = 1.21, 95%CI = 1.09–1.34) quartile were more likely to engage in higher levels of self-reported physical activity outside the school environment. Area level socio-economic status were not associated with physical activity	M
Paquet *et al*. 2014	L	Adults≥18 years N = 3205	neighbourhood walkability, presence of Public Open Spaces (POS) assessed using GIS data	Incidence of pre-diabetes/diabetes, hypertension, dyslipidaemia and abdominal obesity	Australia	Walkability was measured using an index of dwelling density, intersection density, land use entropy and retail foot-print. Increase in walkability score was associated with significant decrease in incidence of prediabetes/diabetes (RR = 0.88 95%CI = 0.80–0.97, P = 0.010). No significant associations were found for hypertension and dyslipidaemia	M
Freedman *et al*. 2008	Not reported	≥ 55 years N = 15,480	Neighbourhood characteristics measured by linking secondary data with GIS data and socio-economic status	Disability	USA	High connectivity of the built environment was associated with reduced risk of limitations in instrumental activities among males (adjusted OR = 0.89, P < 0.05). No association was found between social conditions (immigration, crime and neighbourhood stability) on disability.	M

Berke *et al*. (2007) cross-sectional study reported a significant association between increased neighbourhood walkability and reduced self-reported depressive symptoms among men (OR for IQR of walkability score = 0.31–0.33, P = 0.02). Chiu *et al*. (2016) cohort study assessed the effect of moving from a neighbourhood of low walkability to higher walkability areas on the incidence of hypertension. The authors reported a significantly lower risk of hypertension among people who moved from areas of low walkability to high walkability compared with those who remained in areas of low walkability (Hazard ratio = 0.46, 95%CI = 0.26–0.81, P < 0.01). Findings from the longitudinal study by Paquet *et al*. (2014) showed that an increase in neighbourhood walkability was associated with a significant decrease in the incidence of pre-diabetes/diabetes (RR = 0.88, 95%CI = 0.80–0.97, P = 0.01). James *et al*. (2015) conducted a cohort study to assess the links between neighbourhood walkability and ambient air pollution among women in the United States. The authors reported a positive correlation between neighbourhood walkability and the concentration of PM2.5.

A cross-sectional study to investigate the associations between neighbourhood walkability and BMI found no significant association between higher walk score and BMI or overall obesity. However, people living in highly walkable areas had significantly lower odds of abdominal obesity (waist circumference> 88 cm) compared to counterparts living in less walkable areas (OR = 0.72, 95%CI = 0.53–0.99) (Sriram *et al*. 2016). The study by Mecredy *et al*. (2011) was conducted to evaluate the associations between street connectivity and physical activity among students in 6th to 10th grade across 180 Canadian schools. The findings showed that compared to those living in the highest street connectivity quartile, those in the second (RR = 1.22, 95%CI = 1.110–1.35) third (RR = 1.25, 95%CI = 1.13–1.37) and fourth (RR = 1.21, 95%CI = 1.09–1.34) quartile were more likely to engage in higher levels of self-reported physical activity outside the school environment. Another study by Freedman *et al*. (2008) examined the links between walkability and limitations in carrying out activities of daily living among adults aged 55 and above. The authors reported that high connectivity was associated with a lower risk of having limitations in instrumental activities of daily living among males (OR = 0.89, P < 0.05)

### The impact of access to amenities and public transport

Access to amenities and facilities was found to impact positively on mental wellbeing (Melis *et al*. 2015) and increased physical activity among diverse population groups (Michael *et al*. 2006, Richardsen *et al*. 2016). Richardsen *et al*. (2016) investigated the associations between perceived and objective access to recreational areas and levels of moderate to vigorous physical activity among pregnant women (Table 3). The authors reported that pregnant women residing in neighbourhoods with good access to recreational areas gained nine extra minutes of Moderate to vigorous physical activity (MVPA) per day compared with those living in areas with limited access to recreational facilities (β = 9.14 95%CI = 2.66–15.62 P < 0.01). A randomised-controlled trial investigating the associations between attributes of the neighbourhood and walking among older adults aged 65 and above found that the presence of shopping malls was associated with neighbourhood walking (OR = 4.73, P = 0.035) (Michael *et al*. 2006). A cohort study investigating the impact of density and access to public transport among adults aged 20–64 years found that high urban density (Incidence Rate Ratio (IRR) = 0.92, 95%CI = 0.86–0.97) and improved access to public transport (IRR = 0.93, 95%CI = 0.87–0.98) were associated with lower prescription of anti-depressants among men. Accessibility to public transport was associated with a lower prescription of antidepressants among women of all age groups (Melis *et al*. 2015).

**Table 3.** Main findings from studies on the impact of access to amenities and public transport.

First author, year	Study design^a^	Population	N/hood design Indicator	Health outcome(s)	Location(s)	Main findings	Quality of study
Richardsen *et al*. 2016	Not specified but possibly a Q	Pregnant women, mean age/SD 30.1(4.8) years N = 709	Perceived and objective access to recreational areas in neighbourhood assessed using GIS data	Moderate-to-vigorous physical activity (MVPA)	Norway	. Result shows that women residing in neighbourhoods with good access (objective) to recreational areas gained about 9 additional minutes of MVPA/day compared with those living in areas with limited access to recreational facilities (β = 9.14 95%CI = 2.66–15.62 P < 0.01). In terms of perceived access to recreational areas, perception of high access to recreational areas was associated with 5 additional minutes of MVPA/day compared to areas perceived to have low access (β = 4.75, 95%CI = 0.68–8.82, P = 0.002). The associations reported was not affected by ethnicity or socio-economic status	M
Michael *et al*. 2006	Data was extracted from a RCT	Adults≥65 years N = 105	Characteristics of neighbourhood environment (sidewalk quality, neighbourhood graffiti and vandalism (aesthetics), and presence of shopping malls, parks, and trail) assessed by GIS and an audit system	Walking	USA	After controlling for potential confounders, the presence of a mall was positively associated with neighbourhood walking in the objective model (OR = 4.72, p = .035). No other environmental characteristic showed significant association with walking	M
Melis *et al.* 2015	Cohort	Adults 20–64 years N = 547,263	Urban structure characteristic (density, accessibility by public transport, accessibility to services and public spaces). Data on urban characteristics was retrieved from municipality administrative datasets	Prescription of antidepressants (used as an indicator of mental health)	Italy	After adjusting for some potential confounders, high urban density (Incidence Rate Ratio = 0.92, 95%CI = 0.86–0.97) and high accessibility to public transport (IRR = 0.93, 95%CI = 0.87–0.98) were associated with lower prescription of antidepressants among men age 50–64. Accessibility to public transport was associated with lower prescription of antidepressants among women 20–24 (IRR = 0.94, 95%CI = 0.88–0.99), 34–49 (IRR = 0.95, 95%CI = 0.92–0.99) and 50–64 (IRR = 0.95, 95%CI = 0.92–0.98)	S

### The impact of the quality of the neighbourhood environment

Findings on neighbourhood condition showed significant associations between poor neighbourhood condition and functional loss (Balfour and Kaplan 2002, Schootman *et al*. 2010). Neighbourhood deprivation was also shown to impact negatively on mental wellbeing (Jokela *et al*. 2015) (Table 4).

**Table 4.** Main findings from studies on the impact of quality of the neighbourhood environment.

First author, year	Study design^a^	Population	N/hood design Indicator	Health outcome(s)	Location(s)	Main findings	Quality of study
Balfour and Kaplan 2002	Cohort	Adults ≥ 55 years	traffic, noise, crime, trash and litter, lighting and public transportation	Overall and lower-extremity functional loss	USA	Participants, aged 55 years and older were followed up for one year. 6.1% reported functional loss. Compared with those who did not report problems with neighbourhood environment, those who reported having problems were at higher risks of experiencing overall functional loss (OR = 2.23, 95%CI = 1.08–4.60) and lower-extremity functional loss (OR = 3.12, 95%CI = 1.15–8.51). Inadequate lighting (adjusted OR = 3.20, 95%CI = 1.36–7.56) and excessive noise lighting (adjusted OR = 2.71, 95%CI = 1.38–5.30) showed strong associations with prevalence of self-reported functional loss.	S
Frei *et al*. 2013	Case-C	Adults ≥20 years	Residential distance to high voltage power line	Neurodegenerative conditions	Denmark	The association between Alzheimer’s disease and residency within 50 m of a power line was not statistically significant (Hazard ratio = 1.04; 95%CI = 0.69–1.56). There was an increased risk for persons diagnosed at ages 65–75 (who lived 50 m from a power line) Adjusted hazard ratio = 0.81, 95%CI = 0.95–3.87, but this was not statistically significant.	S
Jokela 2015	Cohort	Adults 16–97 years Mean age (39.5 ± 16.4	Neighbourhood deprivation	General health	UK	Study to investigate the health impact of residential relocation from deprived areas to areas of lower deprivation in England and Wales. Deprivation was measured using the index of multiple deprivation. Neighbourhood deprivation was associated with poorer self-rated health score (OR = 1.34 95%CI = 1.23–1.47), higher psychological distress (OR = 1.18 95%CI = 1.08–1.28), and functional health limitations (OR = 1.40 95%CI = 1.15–1.71).	M
Schootman *et al*. 2010	Cohort	Adults 49–65 years	Neighbourhood conditions (condition of houses, amount of traffic and industry noise, air quality, condition of streets and condition of yards and sidewalks)	Lower-body functional limitations	USA	. Neighbourhood conditions were assessed by 5 markers: condition of houses, amount of traffic and industry noise, air quality, condition of streets and condition of yards and sidewalks. Adjusted model at three year follow up showed that persons with diabetes living in areas rated as fair (OR = 7.79, 95%CI = 1.36–37.55) to poor (OR = 144.6 95%CI = 4.45–775.53) on each of the 5 conditions had higher odds of developing lower-body functional limitations than the referent groups of persons without diabetes who lived in areas rated good-excellent.	M

Aa cohort study to investigate the relationship between markers of neighbourhood quality and functional loss among older adults aged 55 years and above found that participants who self-reported problems with their neighbourhood environment were at higher risks of experiencing overall functional loss (OR = 2.23, 95%CI = 1.08–4.60) and lower-extremity functional loss (OR = 3.12, 95%CI = 1.15–8.51). Inadequate lighting (adjusted OR = 3.20, 95%CI = 1.36–7.56) and excessive noise lighting (adjusted OR = 2.71, 95%CI = 1.38–5.30) showed a strong association with the prevalence of functional loss (Balfour and Kaplan 2002). A case-control study by Frei *et al*. (2013) investigated the associations between residential proximity to a high-voltage power line and the risk of developing Alzheimer’s. The authors found no significant association between the two variables investigated but reported a non-significant increased risk for cases diagnosed between 65 and 75 years. Another cohort study by Jokela (2015) investigated the impact of neighbourhood deprivation on wellbeing among adults. The authors reported that neighbourhood deprivation was associated with poorer self-reported health score (OR = 1.34 95%CI = 1.23–1.47), higher psychological distress (OR = 1.18 95%CI = 1.08–1.28), and functional health limitations (OR = 1.40 95%CI = 1.15–1.71). A study by Schootman *et al*. (2010) was conducted to examine the relationship between living in adverse neighbourhood conditions and the incidence of lower-body functional limitations among adults with diabetes in the US. Neighbourhood condition was assessed by the amount of traffic and industry noise, air quality, the condition of houses, the condition of streets, yards and sidewalks. The authors found that the risk of developing lower-body functional limitations was higher among adults with diabetes living in areas rated as having poor to fair neighbourhood conditions.

## Discussion

This review found some evidence to suggest that the design of the neighbourhood environment is associated with health and wellbeing across all age groups. However, the methodological limitations and study design make it difficult to draw any clear causal links between attributes of the neighbourhood design investigated and health outcomes. Nonetheless, findings from this study demonstrate that access and proximity to green space are associated with a reduced risk factor for cardiovascular diseases, diabetes and respiratory diseases among adults. This is corroborated by findings from a meta-analysis of green space exposure and health outcomes where the authors reported a positive association between exposure to green space and reduced incidence of diabetes, asthma, cardiovascular diseases asthma and all-cause mortality (Twohig-Bennett and Jones 2018). However, the revelation from one of the studies that proximity to green space could be associated with an increased risk of asthma among children should be investigated further (Andrusaityte *et al*. 2016). We also found some evidence to suggest that proximity to green environment could improve levels of physical activity levels. Hartig *et al*. (2014) argued that green space provides an opportunity for physical activity, social cohesion, and stress reduction. The evidence linking neighbourhood green quality and mental health was limited; however, physical activity and social cohesion contribute to positive mental health (van den Berg *et al*. 2019). Co-benefits of neighbourhood greenness on physical activity and mental health was described in one of the studies (Annerstedt *et al*. 2012).

Findings from our review also demonstrate a significant positive association between neighbourhood walkability and various measures of health and wellbeing. Walkability was strongly associated with reduced risk of developing depressive symptoms among men (Berke *et al*. 2007), reduced risk of experiencing limitations in instrumental activities of daily living among men (Freedman *et al*. 2008), reduced incidence of prediabetes and diabetes (Paquet *et al*. 2014). The evidence linking neighbourhood walkability and physical activity was inconsistent. One of the studies in this review reported a negative association between street connectivity and walking among children and adolescents. Those living in the areas ranked as having the highest street connectivity were reported to engage in less time walking than those living in areas ranked as second and third highest street connectivity (Sriram *et al*. 2016). Other studies have reported no association between neighbourhood walkability and leisure time physical activity among various groups (Saelens and Handy 2008, Chudyk *et al*. 2017). This is an area that requires further exploration.

Access to public transport and amenities within the neighbourhood was associated with increased levels of physical activity among several population groups, including older adults and pregnant women. This finding is consistent with previous reviews reporting a positive association between access to amenities on walking and physical activity (Talen and Koschinsky 2013).

Our findings also revealed associations between markers of neighbourhood quality and wellbeing. Markers of neighbourhood quality such as crime, noise, litter, and poor lighting were associated with functional loss (Schootman *et al*. 2010) and functional limitations (Balfour and Kaplan 2002). Neighbourhood deprivation was associated with poor health, psychological distress. Caution should be applied when interpreting these findings as in most cases, the outcome variables were self-reported. Findings from this review also highlight significant gaps in terms of the impact of features of the neighbourhood environment such as safety, connectivity and deprivation on mental wellbeing. However, a systematic review by Truong and Ma (2006) reported associations between neighbourhood deprivation and markers of mental wellbeing. General environmental improvements such as adequate lighting and neighbourhood safety initiatives can reduce the fear of crime (Lorenc *et al*. 2013) and lead to improvements in walking levels (Van Cauwenberg *et al*. 2011).

### Strengths and limitations

One of the main strengths of this review is its clear and systematic approach to the synthesis and appraisal of the quality of all empirical peer-reviewed evidence reporting on the measurable impact of neighbourhood design environment on health and wellbeing at a population level. Findings from the study also highlight areas where there are significant gaps in the evidence base and areas deserving further scrutiny due to inconsistent findings. The evidence provided in this review has the potential to inform the priorities for further research on the neighbourhood environment and health.

This study also has some limitations, which are not exclusive to its design. Only 22 of identified studies (n = 39) were considered to be of moderate or strong quality and included in the review. The majority of the studies deemed to be of weak quality were cross-sectional, lacked objective measures of exposure and outcome variables and included small sample size. The limitations of over-relying on self-reported measures of exposure and outcomes have been well established (Fan *et al*. 2006). The validity and reliability of findings from research studies are determined by the rigour and robustness of the study design. RCTs and other natural experimental designs can produce stronger explanations and inference about causality than observational studies albeit the difficulty in designing experimental studies in the built and natural environment field have been well documented (Gebel *et al*. 2010, Benton *et al*. 2016, Bird *et al*. 2017). Policies and guidelines about the built environment and health should be underpinned by strong and robust evidence (Ige *et al*. 2018). Benton *et al*. (2016) support the argument for evidence-based policy and practice in the built environment and health research domain. The authors reported a contradiction between the quality of studies included in their review and the evidence-based recommendations from a NICE guideline (NICE, 2008). The authors argued that policy recommendations in the field of the built environment and health are often underpinned by inadequate evidence (Benton *et al*. 2016).

## Conclusion

This review identified 39 eligible studies investigating the associations between various features of the neighbourhood environment on health and wellbeing. Our findings broadly strengthen the argument for integrating health and wellbeing into the design of the neighbourhood environment. We also recommend that policymakers in the built environment and health nexus consider not only the evidence of associations or causality but also take into consideration the strengths, weakness, and limitations of the evidence. Policies and guidelines on modifying the built and natural environment should be underpinned by robust evidence, yet despite the abundant literature investigating the impact of several neighbourhood design features on health, the methodological limitations and poor study design of many of these studies give rise to several unanswered questions. Further empirical studies with transparent and clear design are needed to investigate the relationship between neighbourhood greenness and mental health and to understand the associations between neighbourhood walkability and physical activity.

## What this study adds

This study provides a holistic and robust assessment of the associations between all aspects of neighbourhood environment and wellbeing at a population level. This is unlike existing systematic reviews that only consider associations between specific neighbourhood design features (Twohig-Bennett and Jones 2018) on pre-defined health outcomes (Van Cauwenberg *et al*. 2011, Smith *et al*. 2017). The holistic nature of evidence presented in this study supports the consideration of the interactive effects of various design features and outcome measures across the life-course.

The robust approach of identifying and assessing the quality of existing evidence also enabled the identification of research gaps in relation to the nature of evidence in this field. In particular findings from this research provides a rationale for advocating for further research on the impact of neighbourhood design features such as street connectivity, green space and safety on physical and mental wellbeing.

## Appendix

**Included and further excluded studies**

1. Droomers, M., Jongeneel-Grimen, B., Kramer, D., de Vries, S., Kremers, S., Bruggink, J.W., van Oers, H., Kunst, A.E. and Stronks, K., 2016. The impact of intervening in green space in Dutch deprived neighbourhoods on physical activity and general health: results from the quasi-experimental URBAN40 study. *Journal of epidemiology and community health, 70*(2), pp.147–154.
2. Sriram, U., LaCroix, A.Z., Barrington, W.E., Corbie-Smith, G., Garcia, L., Going, S.B., LaMonte, M.J., Manson, J.E., Sealy-Jefferson, S., Stefanick, M.L. and Waring, M.E., 2016. Neighborhood Walkability and Adiposity in the Women’s Health Initiative Cohort. *American journal of preventive medicine*.
3. Picavet, H.S.J., Milder, I., Kruize, H., de Vries, S., Hermans, T. and Wendel-Vos, W., 2016. Greener living environment healthier people?: Exploring green space, physical activity and health in the Doetinchem Cohort Study. *Preventive medicine, 89*, pp.7–14.
4. Pikora, T.J., Giles-Corti, B., Knuiman, M.W., Bull, F.C., Jamrozik, K. and Donovan, R.J., 2006. Neighborhood environmental factors correlated with walking near home: Using SPACES. *Medicine and Science in Sports and Exercise, 38*(4), pp.708–714.
5. Foster, C., Hillsdon, M. and Thorogood, M., 2004. Environmental perceptions and walking in English adults. *Journal of epidemiology and community health, 58*(11), pp.924–928.
6. Ritz, B., Lee, P.C., Hansen, J., Lassen, C.F., Ketzel, M., Sørensen, M. and Raaschou-Nielsen, O., 2016. Traffic-related air pollution and parkinson’s disease in denmark: a case–control study. *Environmental health perspectives, 124*(3), p.351.
7. Melis, G., Gelormino, E., Marra, G., Ferracin, E. and Costa, G., 2015. The effects of the urban built environment on mental health: A cohort study in a large northern Italian city. *International journal of environmental research and public health, 12*(11), pp.14898–14,915.
8. Frei, P., Poulsen, A.H., Mezei, G., Pedersen, C., Salem, L.C., Johansen, C., Röösli, M. and Schüz, J., 2013. Residential distance to high-voltage power lines and risk of neurodegenerative diseases: a Danish population-based case-control study. *American journal of epidemiology*, p.kws334.
9. Egan, M., Kearns, A., Katikireddi, S.V., Curl, A., Lawson, K. and Tannahill, C., 2016. Proportionate universalism in practice? A quasi-experimental study (GoWell) of a UK neighbourhood renewal programme’s impact on health inequalities. *Social Science & Medicine, 152*, pp.41–49.
10. Richardsen, K.R., Mdala, I., Berntsen, S., Ommundsen, Y., Martinsen, E.W., Sletner, L. and Jenum, A.K., 2016. Objectively recorded physical activity in pregnancy and postpartum in a multi-ethnic cohort: association with access to recreational areas in the neighbourhood. *International Journal of Behavioral Nutrition and Physical Activity, 13*(1), p.1.
11. James, P., Hart, J.E. and Laden, F., 2015. Neighborhood walkability and particulate air pollution in a nationwide cohort of women. *Environmental research, 142*, pp.703–711.
12. Wells, N.M. and Yang, Y., 2008. Neighborhood design and walking: a quasi-experimental longitudinal study. *American journal of preventive medicine, 34*(4), pp.313–319.
13. Schootman, M., Andresen, E.M., Wolinsky, F.D., Miller, J.P., Yan, Y. and Miller, D.K., 2010. Neighborhood conditions, diabetes, and risk of lower-body functional limitations among middle-aged African Americans: a cohort study. *BMC public health, 10*(1), p.1.
14. Chiu, M., Rezai, M.R., Maclagan, L.C., Austin, P.C., Shah, B.R., Redelmeier, D.A. and Tu, J.V., 2016. Moving to a highly walkable neighborhood and incidence of hypertension: a propensity-score matched cohort study. *Environmental Health Perspectives (Online), 124*(6), p.754.
15. Annerstedt, M., Östergren, P.O., Björk, J., Grahn, P., Skärbäck, E. and Währborg, P., 2012. Green qualities in the neighbourhood and mental health–results from a longitudinal cohort study in Southern Sweden. *BMC public health, 12*(1), p.1.
16. Paquet, C., Coffee, N.T., Haren, M.T., Howard, N.J., Adams, R.J., Taylor, A.W. and Daniel, M., 2014. Food environment, walkability, and public open spaces are associated with incident development of cardio-metabolic risk factors in a biomedical cohort. *Health & place, 28*, pp.173–176.
17. Shepherd, M., Austin, P. and Chambers, J., 2010. Driveway runover, the influence of the built environment: a case control study. *Journal of paediatrics and child health, 46*(12), pp.760–767.
18. Jokela, M., 2015. Does neighbourhood deprivation cause poor health? Within-individual analysis of movers in a prospective cohort study. *Journal of epidemiology and community health*, pp.jech-2014.
19. Andrusaityte, S., Grazuleviciene, R., Kudzyte, J., Bernotiene, A., Dedele, A. and Nieuwenhuijsen, M.J., 2016. Associations between neighbourhood greenness and asthma in preschool children in Kaunas, Lithuania: a case–control study. *BMJ open, 6*(4), p.e010341.
20. Tamosiunas, A., Grazuleviciene, R., Luksiene, D., Dedele, A., Reklaitiene, R., Baceviciene, M., Vencloviene, J., Bernotiene, G., Radisauskas, R., Malinauskiene, V. and Milinaviciene, E., 2014. Accessibility and use of urban green spaces, and cardiovascular health: findings from a Kaunas cohort study. *Environmental Health, 13*(1), p.1.
21. Villeneuve, P.J., Jerrett, M., Su, J.G., Burnett, R.T., Chen, H., Wheeler, A.J. and Goldberg, M.S., 2012. A cohort study relating urban green space with mortality in Ontario, Canada. *Environmental research, 115*, pp.51–58.
22. Addy, C.L., Wilson, D.K., Kirtland, K.A., Ainsworth, B.E., Sharpe, P. and Kimsey, D., 2004. Associations of perceived social and physical environmental supports with physical activity and walking behavior. *American journal of public health, 94*(3), pp.440–443.
23. Ainsworth, B.E., Wilcox, S., Thompson, W.W., Richter, D.L. and Henderson, K.A., 2003. Personal, social, and physical environmental correlates of physical activity in African-American women in South Carolina. *American journal of preventive medicine, 25*(3), pp.23–29.
24. Balfour, J.L. and Kaplan, G.A., 2002. Neighborhood environment and loss of physical function in older adults: evidence from the Alameda County Study. *American Journal of Epidemiology, 155*(6), pp.507–515.
25. Berke, E.M., Gottlieb, L.M., Moudon, A.V. and Larson, E.B., 2007. Protective association between neighborhood walkability and depression in older men. *Journal of the American Geriatrics Society, 55*(4), pp.526–533.
26. Cohen, D.A., Golinelli, D., Williamson, S., Sehgal, A., Marsh, T. and McKenzie, T.L., 2009. Effects of park improvements on park use and physical activity: policy and programming implications. *American journal of preventive medicine, 37*(6), pp.475–480.
27. De Meester, F., Van Dyck, D., De Bourdeaudhuij, I., Deforche, B., Sallis, J.F. and Cardon, G., 2012. Active living neighborhoods: is neighborhood walkability a key element for Belgian adolescents?. *BMC public health, 12*(1), p.1.
28. Deshpande, A.D., Baker, E.A., Lovegreen, S.L. and Brownson, R.C., 2005. Environmental correlates of physical activity among individuals with diabetes in the rural midwest. *Diabetes Care, 28*(5), pp.1012–1018.
29. Freedman, V.A., Grafova, I.B., Schoeni, R.F. and Rogowski, J., 2008. Neighborhoods and disability in later life. *Social science & medicine, 66*(11), pp.2253–2267.
30. Giles-Corti, B., Broomhall, M.H., Knuiman, M., Collins, C., Douglas, K., Ng, K., Lange, A. and Donovan, R.J., 2005. Increasing walking: how important is distance to, attractiveness, and size of public open space?. *American journal of preventive medicine, 28*(2), pp.169–176.
31. Grafova, I.B., Freedman, V.A., Kumar, R. and Rogowski, J., 2008. Neighborhoods and obesity in later life. *American Journal of Public Health, 98*(11), pp.2065–2071.
32. Michael, Y.L., Beard, T., Choi, D., Farquhar, S. and Carlson, N., 2006. Measuring the influence of built neighborhood environments on walking in older adults.
33. Prince, S.A., Kristjansson, E.A., Russell, K., Billette, J.M., Sawada, M.C., Ali, A., Tremblay, M.S. and Prud’homme, D., 2012. Relationships between neighborhoods, physical activity, and obesity: a multilevel analysis of a large Canadian city. *Obesity, 20*(10), pp.2093–2100.
34. West, S.T. and Shores, K.A., 2015. Does building a greenway promote physical activity among proximate residents?. *Journal of physical activity & health, 12*(1).
35. Owen, N., Cerin, E., Leslie, E., Coffee, N., Frank, L.D., Bauman, A.E., Hugo, G., Saelens, B.E. and Sallis, J.F., 2007. Neighborhood walkability and the walking behavior of Australian adults. American journal of preventive medicine, 33(5), pp.387–395.
36. Van Herzele, A. and de Vries, S., 2012. Linking green space to health: a comparative study of two urban neighbourhoods in Ghent, Belgium. Population and Environment, 34(2), pp.171–193.
37. Sugiyama, T., Thompson, C.W. and Alves, S., 2009. Associations between neighborhood open space attributes and quality of life for older people in Britain. Environment and Behavior, 41(1), pp.3–21.
38. Sugiyama, T., Francis, J., Middleton, N.J., Owen, N. and Giles-Corti, B., 2010. Associations between recreational walking and attractiveness, size, and proximity of neighborhood open spaces. American Journal of Public Health, 100(9), pp.1752–1757.
39. Mueller, N., Rojas-Rueda, D., Basagaña, X., Cirach, M., Cole-Hunter, T., Dadvand, P., Donaire-Gonzalez, D., Foraster, M., Gascon, M., Martinez, D. and Tonne, C., 2016. Urban and transport planning related exposures and mortality: a health impact assessment for cities. Environ. Health Perspect. in print.
  **Table:**
  Further excluded studies
  Kingsbury, M., Kirkbride, J.B., McMartin, S.E., Wickham, M.E., Weeks, M. and Colman, I., 2015. Trajectories of childhood neighbourhood cohesion and adolescent mental health: evidence from a national Canadian cohort. *Psychological medicine, 45*(15), pp.3239–3248. Gern, J.E., Visness, C.M., Gergen, P.J., Wood, R.A., Bloomberg, G.R., T O’Connor, G., Kattan, M., Sampson, H.A., Witter, F.R., Sandel, M.T. and Shreffler, W.G., 2009. The Urban Environment and Childhood Asthma (URECA) birth cohort study: design, methods, and study population. *BMC pulmonary medicine, 9*(1), p.1. Bosma, H., van de Mheen, H.D., Borsboom, G.J. and Mackenbach, J.P., 2001. Neighborhood socioeconomic status and all-cause mortality. *American Journal of Epidemiology, 153*(4), pp.363–371. Roux, A.V.D., Borrell, L.N., Haan, M., Jackson, S.A. and Schultz, R., 2004. Neighbourhood environments and mortality in an elderly cohort: results from the cardiovascular health study. *Journal of epidemiology and community health, 58*(11), pp.917–923. Astell-Burt, T., Feng, X. and Kolt, G.S., 2016. Large-scale investment in green space as an intervention for physical activity, mental and cardiometabolic health: study protocol for a quasi-experimental evaluation of a natural experiment. *BMJ open, 6*(4), p.e009803. Jamshidi, E., Moradi, A. and Majdzadeh, R., 2016. Environmental risk factors contributing to traffic accidents in children: a case-control study. *International Journal of Injury Control and Safety Promotion*, pp.1–7. Newbury, J., Arseneault, L., Caspi, A., Moffitt, T.E., Odgers, C.L. and Fisher, H.L., 2016. Why are Children in Urban Neighborhoods at Increased Risk for Psychotic Symptoms? Findings From a UK Longitudinal Cohort Study. *Schizophrenia bulletin*, p.sbw052.
